# Replacing the eleven native tryptophans by directed evolution produces an active P-glycoprotein with site-specific, non-conservative substitutions

**DOI:** 10.1038/s41598-020-59802-w

**Published:** 2020-02-21

**Authors:** Douglas J. Swartz, Anukriti Singh, Narong Sok, Joshua N. Thomas, Joachim Weber, Ina L. Urbatsch

**Affiliations:** 1 From the Department of Cell Biology and Biochemistry, Texas Tech University Health Sciences Center, Lubbock, Texas USA; 2 Center for Membrane Protein Research, Texas Tech University Health Sciences Center, Lubbock, Texas USA; 3 Department of Chemistry and Biochemistry, Texas Tech University, Lubbock, Texas USA; 4 Present Address: Department of Natural Sciences, Lubbock Christian University, Lubbock, TX 79407 USA

**Keywords:** Carrier proteins, Membrane proteins, Protein design

## Abstract

P-glycoprotein (Pgp) pumps an array of hydrophobic compounds out of cells, and has major roles in drug pharmacokinetics and cancer multidrug resistance. Yet, polyspecific drug binding and ATP hydrolysis-driven drug export in Pgp are poorly understood. Fluorescence spectroscopy using tryptophans (Trp) inserted at strategic positions is an important tool to study ligand binding. In Pgp, this method will require removal of 11 endogenous Trps, including highly conserved Trps that may be important for function, protein-lipid interactions, and/or protein stability. Here, we developed a directed evolutionary approach to first replace all eight transmembrane Trps and select for transport-active mutants in Saccharomyces cerevisiae. Surprisingly, many Trp positions contained non-conservative substitutions that supported *in vivo* activity, and were preferred over aromatic amino acids. The most active construct, W(3Cyto), served for directed evolution of the three cytoplasmic Trps, where two positions revealed strong functional bias towards tyrosine. W(3Cyto) and Trp-less Pgp retained wild-type-like protein expression, localization and transport function, and purified proteins retained drug stimulation of ATP hydrolysis and drug binding affinities. The data indicate preferred Trp substitutions specific to the local context, often dictated by protein structural requirements and/or membrane lipid interactions, and these new insights will offer guidance for membrane protein engineering.

## Introduction

Pgp (also known as multidrug resistance protein, MDR1 or ABCB1) is a plasma membrane protein with the ability to pump a wide range of hydrophobic substances out of cells using the energy of ATP hydrolysis^1–3^. It is important in determining the pharmacokinetics of drugs by extruding them from cells and by participating in transepithelial transport of drugs and metabolites^4,5^. Pgp substrates include many drugs used for treatment of cancer, HIV/AIDS, neurodegenerative and cardiovascular diseases, as well as herbal supplements (St. John’s wort)^6–13^. Pgp is expressed in many human tissues, including the intestinal and blood-brain barriers, liver and kidneys, where it has a physiological role in protecting the body from xenobiotic toxins, by limiting their absorption and enhancing their excretion, respectively^14–17^. Its importance in drug bioavailability and pharmacokinetics is well recognized by the US Food and Drug Administration (FDA) and the European Medicines Agency (EMA) who now require drug interaction studies with Pgp for approval of any new drug^18–21^. Consequently, there is great interest in understanding the mechanism by which drugs interact with Pgp, and in developing new drug-binding assays.

Understanding how Pgp accomplishes polyspecific substrate binding and how binding modulates Pgp function is necessary for structure based Pgp drug design. Pgp is an ATP binding cassette (ABC) transporter containing two transmembrane domains (TMDs) that harbor the drug binding sites, and two cytoplasmic nucleotide binding domains (NBDs) that bind and hydrolyze ATP. Classical competitive and noncompetitive substrate binding interactions were first observed in photolabelling experiments using photoreactive substrate analogs^22–25^. Radioligand saturation and displacement assays have identified at least four pharmacologically distinct substrate binding sites within Pgp that are linked by negative or positive allosteric interactions^26,27^ (reviewed in^24,28^). Positively cooperative sites for drug transport were observed for the fluorescent substrates Hoechst and Rhodamine (H and R sites)^29,30^, while binding of prazosin (P site) modulated transport at the H and R sites. Extensive attempts have been made to identify residues in the transmembrane helices by cysteine-scanning mutagenesis that can be labeled with thiol-reactive drug substrates and/or are protected by certain drugs from labeling, as well as those that can be protected by certain drugs from Cys-Cys-crosslinking of adjacent transmembrane helices^31,32^. X-ray crystallography and cryo-electron microscopy (EM) structures show one or two ligands bound to distinct but sometimes overlapping sites within a large binding surface in the center of the TMDs^33–38^. Complex competitive and mixed-type inhibition were also observed in drug-stimulated ATPase assays suggesting positional overlap of classical transport substrates with ligands localized in the structures^39^. The structures have motivated a flurry of site directed mutagenesis studies to probe contact residues for binding of cancer drugs and classical substrates^32,40–42^ (reviewed in^3^) as well as other inhibitors for which structures are not yet available, supported by molecular dynamics and docking simulations^43–46^. The emerging picture is that of spatially distinct binding sites that can simultaneously bind multiple drugs, as well as spatially overlapping binding sites for certain drugs that are linked through cooperative and non-cooperative interactions^3,41,42,45^. Nevertheless, more work is needed to determine the physical locations of those drug binding sites in Pgp and the spatial interactions of therapeutic drugs and inhibitors. Similarly, the mechanism by which ATP binding and hydrolysis achieve the conformational changes necessary to expel drugs from the cell is only partially understood^47–50^.

**Figure 1:**
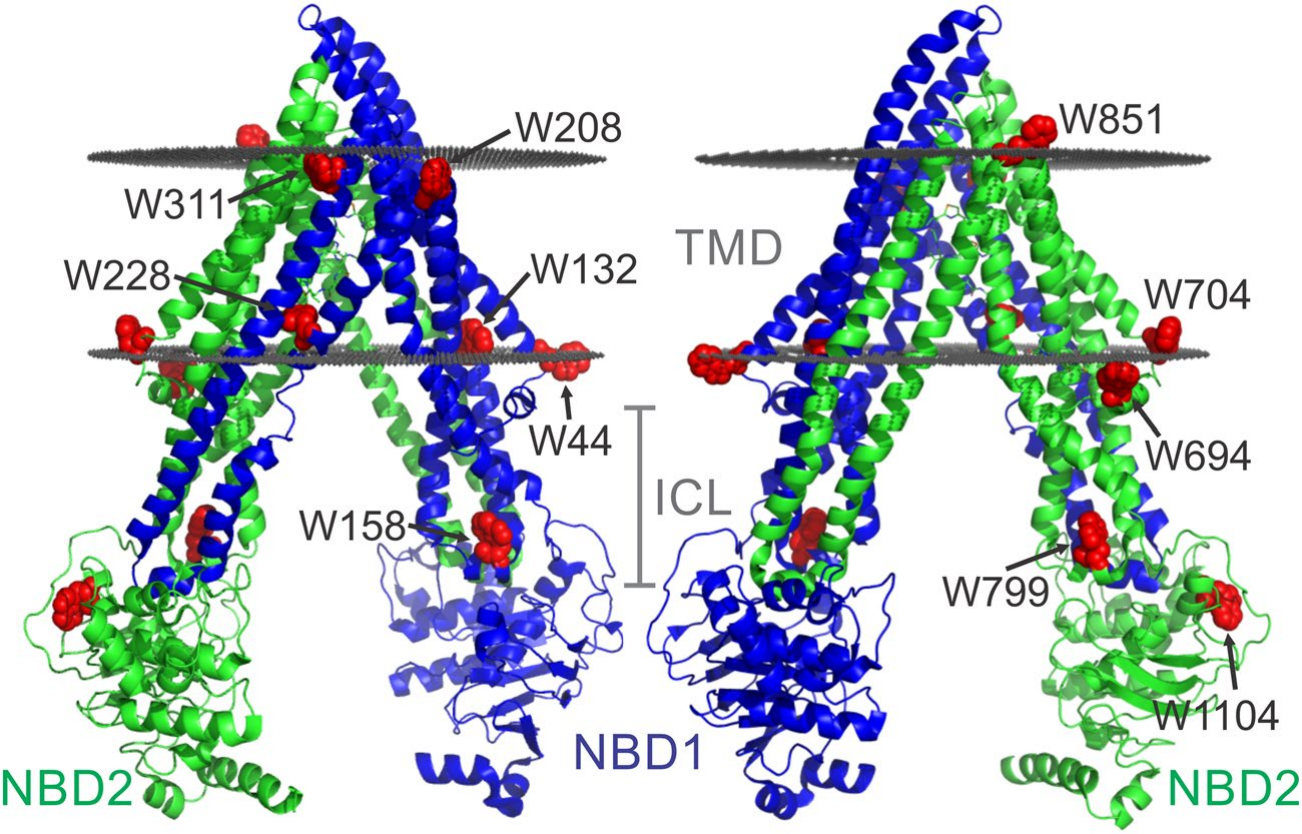
Locations of the eleven native Trp residues in Pgp. Crystal structure of Pgp (PDB ID: 4QH9)^36^ with the N- and C-terminal halves of the protein colored in blue and green, respectively. Pgp has eight Trps in the transmembrane domains (TMDs), and three Trps in the cytoplasmic domains shown as red spheres and labeled with residue numbers. Shaded gray discs indicate the approximate boundaries of the hydrophobic core of the lipid bilayer downloaded from the Orientation of Proteins in Membranes database (http://opm.phar.umich.edu/)^114,115^. ICL, intracellular loop.

A well-established technique to study ligand binding to a protein and the associated conformational changes is Trp fluorescence spectroscopy, due to the pronounced sensitivity of Trp fluorescence to changes in the environment of the fluorophore. Pgp has eleven intrinsic Trps, which have been used to investigate binding of ATP as well as drugs; these studies were limited to small changes in Trp fluorescence upon binding of some ligands (<10% quenching by ATP), and could not localize a discrete site(s) for drug binding^51–54^. However, the native Trps are not ideally located (Fig. 1). One Trp (W228) resides in the hydrophobic core of the lipid bilayer, in proximity of a known drug binding site^36,55^. Seven more Trps are located at the membrane water interface; W208, W311 and W851 are located in extracellular loops close to the outer membrane interface, while W132 is at the cytoplasmic membrane interface along with W44, W694, and W704, located in the elbow helices that parallel the membrane. The three remaining Trps are located in the intracellular portions of the protein, distal to where drug binding occurs in the TMDs. W158 and W799 are located within the intracellular loops (ICLs) that extend from the TMDs and make contact with the NBDs, and W1104 is in the C-terminal NBD (NBD2).

A better approach is the use of site-specific Trp fluorescence, where Trp residues are inserted close to the site(s) of interest, in the case of Pgp the binding sites for drugs and ATP. For this approach, however, the background fluorescence of the 11 native Trps presents a nearly insurmountable obstacle. In order to fully exploit the advantages of site-specific Trp fluorescence, the intrinsic Pgp fluorescence must be reduced or eliminated before strategically placed Trps can be used to localize the discrete drug binding sites and to measure binding affinities for drugs and ATP. A number of proteins^56–66^, including some membrane proteins^67–72^, have been rendered Trp-free, requiring the removal of between 1 and 13 Trp residues. In most cases, Trp was replaced by one of the other aromatic amino acid residues, Tyr or Phe; occasionally, Leu was used.

The first attempt to create Trp-free Pgp by replacing all 11 Trps by Phe (“All-F”)^73^ gave a transporter with minimal function and impaired expression, making it unusable as a tool for further studies. Subsequent experiments showed that multiple Pgp Trps could be successfully removed, but also suggested that aromatic amino acids may not be the ideal substitute at several Pgp Trp locations^74^. In the context of a large integral membrane protein, Trps can be found in a variety of local environments, as demonstrated by Pgp. Trps can be buried within the lipid bilayer, reside at the inner or outer membrane water interface, or within the water-soluble portions of the protein, suggesting that one amino acid may not always be the best Trp replacement. In the present study, we describe the development and the use of a directed evolution procedure for replacing the endogenous Pgp Trps. Directed evolution is the process of selecting for traits that are not found in nature, and has been used to engineer proteins with specific properties, such as thermostability, enantioselectivity, and high affinity antibodies. In this study, we designed a directed evolution strategy to select for active Trp-free Pgp proteins. Site-saturation mutagenesis replaced Pgp Trps with every other amino acid. Then, the directed evolution constructs were expressed in *S. cerevisiae* for selection of active Trp mutants by complementation for Ste6, a homologous pheromone transporter required for yeast mating, and the ability to convey fungicide resistance to the yeast. The Pgp Trps were replaced in three blocks; initially the three outer membrane Trps plus W228 were simultaneously replaced, and then the active mutants were used as a template to replace the four inner membrane Trps to account for intradomain interactions. The combined full-length Pgp mutants (with eight Trps replaced) were retransformed into naïve yeast and subjected to a second round of screening to select the most active mutant combinations. One of the most active mutant combinations (W(3Cyto)) was chosen as a template to replace the three cytoplasmic Trps and create Trp-less (WL)-Pgp. Surprisingly, directed evolution revealed a large bias towards non-conservative Trp mutations at some positions. These results suggest that determining the ‘best’ amino acid substitution for a residue in a membrane bound transporter is highly dependent on the local environment of the residue. Functional integrity of the most active W(3Cyto) and WL-Pgp were scrutinized *in vivo* by drug resistance and cellular localization studies, and in the purified proteins by ATPase assays, protein thermostability and Trp fluorescence spectroscopy.

## Results

### Trp mutant library construction and screening

**Figure 2:**
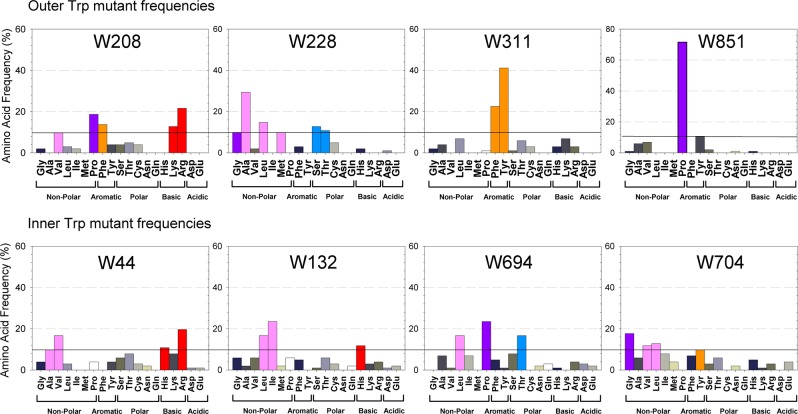
Directed evolution reveals non-traditional substitutions of transmembrane Trps. Distribution of amino acid substitutions that successfully mated found for the outer Trp block mutants (top), and for the inner Trp block mutants (bottom). Amino acid frequencies were calculated from a total of 102 mutants (72 unique mutants) that successfully mated and showed resistance to doxorubicin and FK506. Amino acids occurring at a frequency of >10% were color coded according to the Zappo scheme^116^.

The first objective of this study was to replace all eight TMD Trps by site-saturation mutagenesis, allowing every possible amino acid substitution at every Trp position, and to determine which amino acid combinations permit a fully active Pgp. Ideally, all eight native Trps would be replaced simultaneously to account for potential interactions among Trp substitutions. However, the extremely large number of possible combinations (19^8^ = 1.7 × 10^10^) makes that approach impractical. Instead, we replaced the Trps in two sequential blocks of four simultaneous Trp substitutions, reducing the required number of mutants (19^4^ = 130,321 combinations per block of 4). The first block contained W208, W311, and W851 in the outer leaflet plus W228 in the inner vestibule (named “outer Trp” block for short). The second block contained the four Trps in the inner leaflet, W44, W694 and W704 in the elbow helices, and W132 (named “inner Trp” block). For site-saturation mutagenesis, an overlap-extension PCR approach was used to replace the native Trps with degenerate primer pairs that encode either all 20 amino acids (64 codons) or a mixture of oligos encoding all amino acids except Trp (devoid of TGG, see Methods), then the fragments assembled by overlap extension PCR, as outlined in Supplemental Figure S1A. The mutant PCR libraries were directly transformed into *S. cerevisiae* via homologous recombination (see General approach, Fig. 2) to select for active mutants that retained their ability to complement for Ste6, a Pgp yeast homologue, and export **a**-factor pheromone required for mating (a farnesylated dodecapeptide YIIKGVFWDPAC(S-farnesyl)OCH_3_)^75–77^. Positive clones were then screened in two different fungicidal drugs, FK506 and doxorubicin. This selection scheme was designed to identify mutants that could preserve polyspecific drug transport, an important quality of Pgp, based on its ability to export the **a**-mating factor and convey fungicidal resistance to yeast against two drugs^74,78,79^. The blocks from clones that mated and were active in both drug assays were sequenced to identify Trp-free clones.

In total, approximately 300,000 transformants carrying the outer Trp block entered the selection process, and we identified 316 unique, active Trp-free mutants (Table 1). Plasmid DNA from these mutants was extracted from yeast, PCR amplified, and pooled to serve as a template for site saturation mutagenesis of the inner Trp block. A total of 272 amplicons were obtained from the outer block and used as template to construct the inner block. A PCR mutant library for the inner Trp block, was assembled as outlined in Supplemental Figure S1B, transformed into yeast, mated, screened for drug resistance, and colonies sequenced across the four newly introduced Trp substitutions. Screening of over 200,000 colonies initially yielded 196 mutants with the four inner leaflet Trps substituted. These 196 plasmids were extracted from yeast, retransformed into naïve JPY201 yeast, and extensively screened in mating and drug resistance assays., Concentrations of drugs used for selection (50 µg/ml FK506 and 30 µM doxorubicin) allowed clear discernment between active and inactive mutants, with negative controls (“vector”) exhibiting less than 15% of the growth of Wt Pgp in these assays (see Methods for details). Plasmid DNAs were then resequenced across the four outer Trp and the four inner Trp block substitutions. This strategy identified 72 active unique mutants (102 total mutants) with all eight TMD Trps substituted.

**Table 1:** Directed Evolution.

	Transformants	Colonies screened for drug resistance	Colonies sequenced	Trp-free	Re-screened in naïve yeast
Outer Trps	300,000	1,116	624	316	not done
Outer and Inner Trps	200,000	1,143	768	196	72 unique^a^ (102 total)
Cytoplasmic Trps	500,000	—	447	256	9 unique (15 total)

^a^For this study, unique mutants are defined as a distinct combination of amino acids with distinct codons; some combinations occurred more than once.

### Trends of Trp substitutions found by directed evolution

Frequencies of amino acid substitutions for each Trp of the outer Trp and inner Trp blocks are given in Fig. 2. Preferred substitutions vary and appear specific to the location of Trp within the protein. Notably, W311 was the only position that favored the traditional aromatic substitutions Phe and Tyr (Fig. 2, top center). Surprisingly, W851 exhibited a very strong preference for Pro. Its counterpart in TMD1, W208, also displayed preference for Pro and Phe, but also accommodated positively charged Lys and Arg. W228 tolerated a variety of substitutions, such as small (Ala, Gly) or larger (Leu, Met) hydrophobic residues and the relatively small polar Ser and Thr. Of the four Trp residues grouped together in the inner Trp block, W694 and W704 allowed a spectrum of different substitutions, Pro, Leu and Thr for W694, and Gly, Val, Leu and Tyr for 704. Positions W44 and W132 showed a preference for hydrophobic and positively charged residues. The screen results are interesting in that they suggest regions of greater stringency (W851 and W311), and may provide guidance for substitutions of Trp in other membrane transporter proteins. A more detailed analysis follows in the Discussion.

### Active combinations of substitutions of all eight TMD Trps

**Figure 3:**
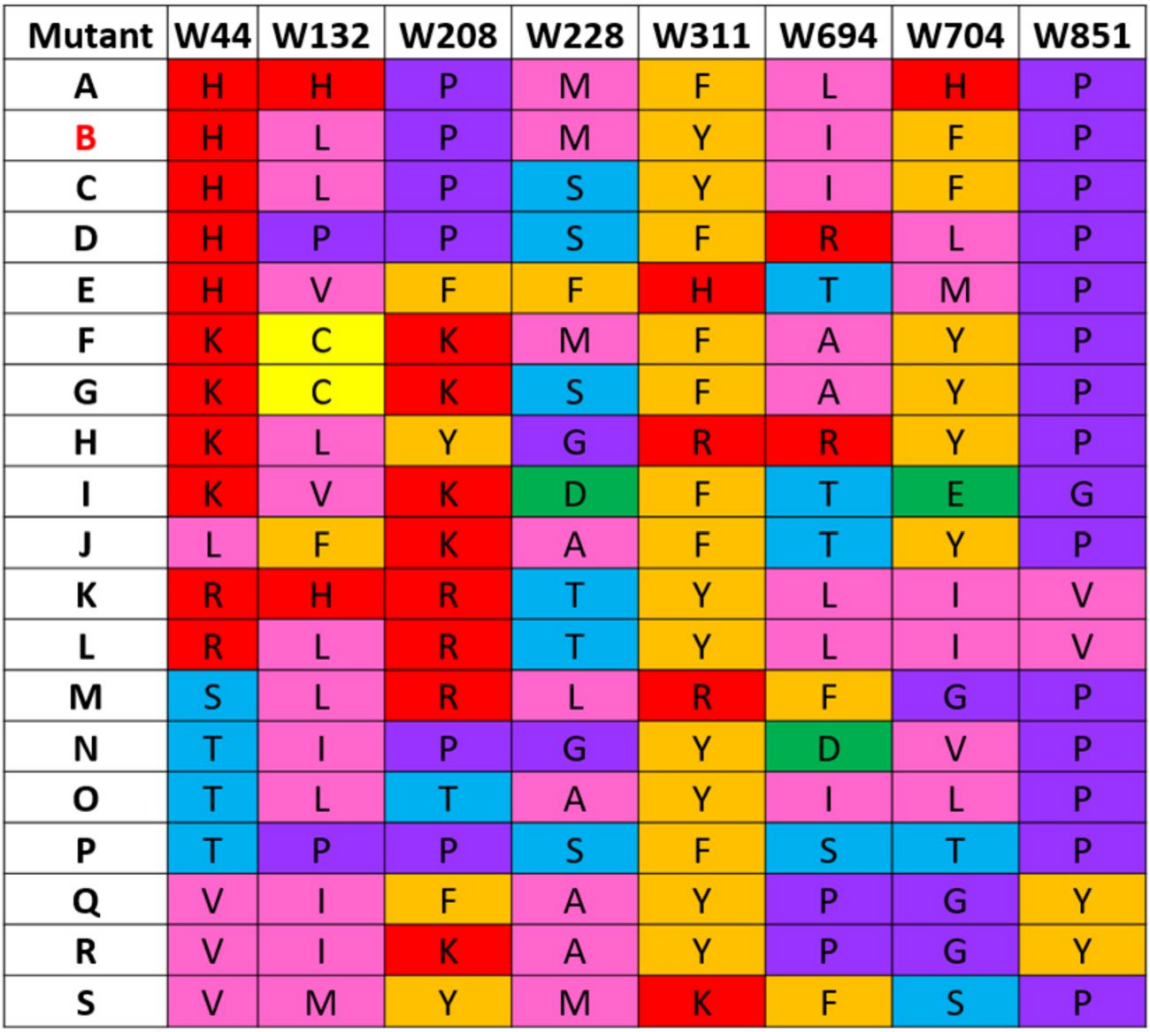
Identity of mutants with all eight transmembrane Trps replaced. Identity of amino acid substitutions found at each of the 8 transmembrane Trp positions in the 19 most active mutant combinations. Apparent trends and patterns were color coded by side chain classification according to the Zappo scheme^116^. “Mutant B” was named W(3Cyto), and was chosen for further studies.

Iterative screening in naïve yeast of the 72 unique mutants, with the eight Trps in the transmembrane region replaced, gave the 19 most active mutant combinations shown in Fig. 3. The most active in all three assays were ‘mutant B’ and ‘mutant C’ that retained wild-type like activities in mating (101 ± 15%, 81 ± 13%, respectively), and resistance to FK506 (77 ± 6%, 68 ± 11%) and to doxorubicin (52 ± 14%, 43 ± 13%). Interestingly, mutant B and C did NOT contain all of the most prominent substitutions found at each of the eight Trp positions (Fig. 2). They did contain the most frequent substitution at positions W311Y and W851P, and the second most frequent substitutions at W132L and W208P. In addition, they contained substitutions that occurred at least with 10% frequency at positions W44H and W228M/S, while substitutions W694I and W704F are present with only 8% frequency among all mutants (Fig. 2). The data indicate crosstalk between residues within the TMDs. Thus, our strategy to first select the 316 active outer Trp block mutants, and then use those as templates to build the inner Trp block mutants proved successful. Our yeast screen served as a very powerful means to disclose the few specific combinations of substitutions that preserve function. ‘Mutant B’ with all eight transmembrane Trps substituted, W44H/W132L/W208P/W228M/W311Y/W694I/W704I/W851P, was named W(3Cyto) and was chosen for further studies.

### Trp-free Pgp

**Figure 4:**
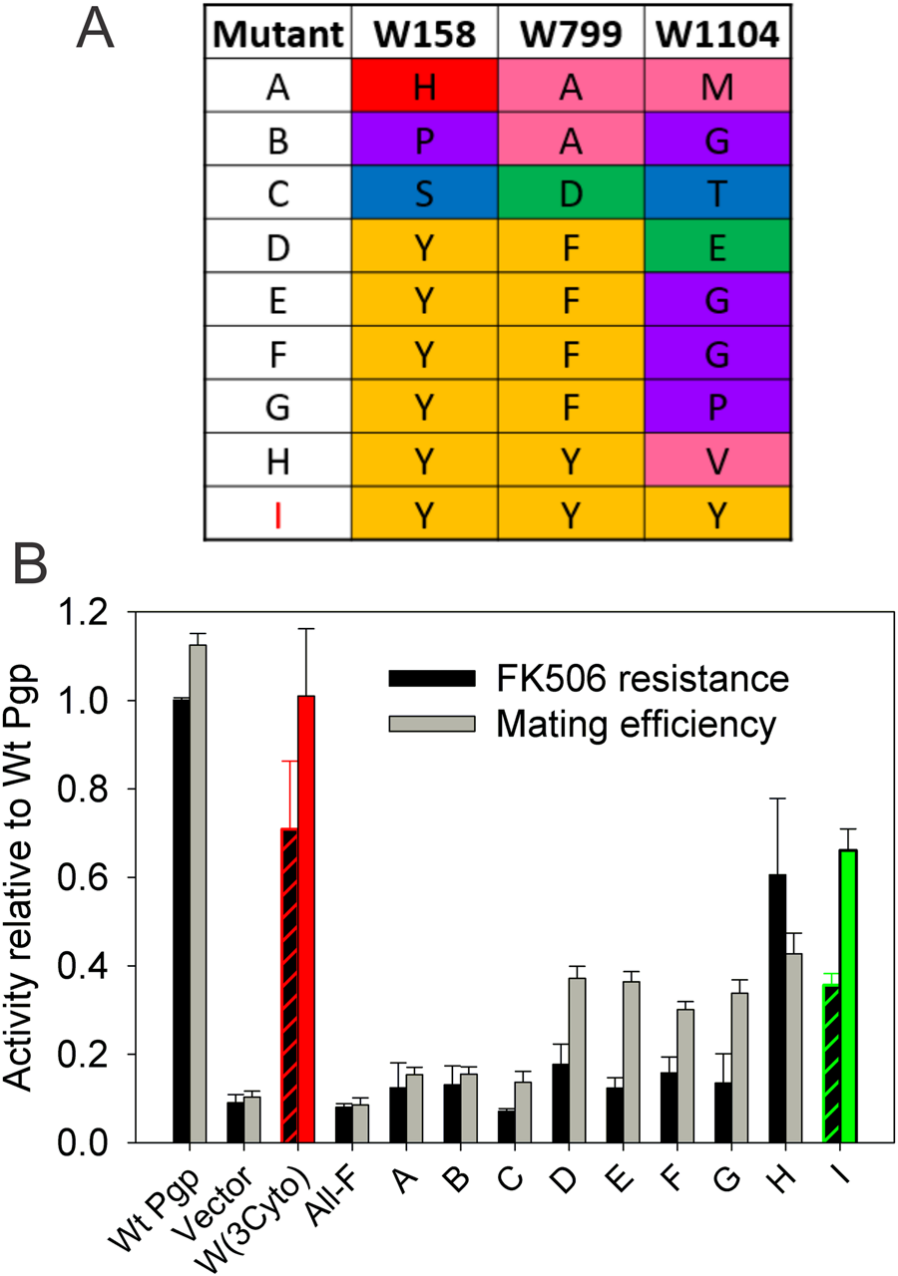
Identity and activity of a subset of mutants with all 11 Trps replaced. (**A**) Identity of amino acid substitutions found at each of the three cytoplasmic Trp positions in the 9 most active Trp-free mutant combinations. Apparent trends and patters were color coded in Zappo^116^ as in Fig. 4. (**B**) *In vivo* activity of Trp-less mutants. Mating efficiency was determined by the number of diploid yeast colonies relative to the total number of colonies, and compared to Wt Pgp expressing yeast. To assess drug resistance yeast transformants were grown in the presence of 50 μg/ml FK506 for 30 hrs, and the optical density of the mutant cells and compared to yeast expressing Wt Pgp (gray bars). Bars represent the mean of ≥3 independent experiments ± SEM. Mutant I (green box) was chosen for further studies.

The main objective of this study was to generate an active Trp-free Pgp. W(3Cyto) was used as the template to replace the three remaining cytoplasmic Trps. Again, directed evolution allowed every possible amino acid substitution at every Trp position, and determined which amino acid combinations could generate a fully active Pgp. Replacing three Trps with every other amino acid would require 19^3^ = 6,859 possible combinations. Therefore, it was surprising that more than 500,000 transformants had to be screened to produce 346 of potential mutant colonies, as very few of the yeast transformants retained mating activity. Colony PCR sequencing revealed 265 of these colonies were Trp free at both W158 and W799, which are both in key positions in the coupling helices connecting the TMDs and NBDs. W1104, located in the backbone of NBD2 could be substituted by many amino acid and was not routinely sequenced during the initial mutant identification. Mutant plasmids lacking Trp at W158 and W799 were extracted, retransformed into naïve yeast, and the mutants screened again for their ability to mate. Mating activity was very low for a majority of the mutants resulting in a final set of 22 mutants that were full length sequenced. 9 unique Trp-free mutants were identified and studied in more detail for mating efficiency and drug resistance (Fig. 4). In these *in vivo* activity assays, a previously generated Trp-free Pgp mutant was included that has all 11 Trps replaced by the aromatic Phe^73^. That “All F” Pgp mutant showed very low mating efficiency and little to no drug resistances in yeast cells (Fig. 4B). We found that mating efficiency and resistance to FK506 was low for most of the mutants, similar to the “All F” Pgp mutant. For example, mutants A, B, and C in Fig. 4 showed a relative mating efficiency <0.1, barely higher than vector control samples that do not express Pgp, as well as low FK506 resistance (<20% of WT activity). Mutants D, E, F and G showed somewhat higher mating efficiency (30 to 40%) but drug resistance was still low (<20% of WT activity). Of the active nine unique (11 total) mutants with the three cytoplasmic Trps substituted shown in Fig. 4A, only two mutants, ‘Mutant H’ and ‘Mutant I’ retained good activity in both mating efficiency and drug resistance against FK508 (Fig. 4B); the latter was obtained three times from independent transformations/selections.

In positions W158 and W799, we had previously tested single substitutions W158F and W799F in a wild type Pgp background and found almost complete loss of mating activity^74^. Here, when fully degenerate primers were used for the mutagenesis (“Trp+”; see Materials and Methods), these were the only two positions that retained the Trp in the vast majority of cases. Substitutions at position W158 showed a clear preference for Tyr (8 out of 11 clones), while at W799 both Tyr and Phe (4 and 4 out of 11 total clones) were found. However, Tyr substitution at W799 (Mutants H and I) were significantly more active than Phe substitutions (Mutants D, E, F and G). Finally, substitutions at W1104 were more random with three Gly, three Tyr, and one Glu, Pro, Val, Met and Thr observed in 15 independent clones; the two most active mutants contained Val or Tyr at this position (Fig. 4, Mutants H and I). It is possible that this higher activity is caused by the fact that the mutants with Val and Tyr in position 1104 had a Tyr and not a Phe in position 799.

Taken together, directed evolution was able to identify not just the best substitutions for each of the 11 Trp positions, but revealed two unique combinations of substitutions that maintained high activity. Mutant I with all eleven Trps substituted (W44H/W132L/W208P/W228M/W311Y/W694I/W704I/W851P plus W158Y/W799Y/W1104Y, green box in Fig. 4B) was named Trp-less (WL-Pgp).

### *In-vivo* analyses of W(3Cyto) and WL-Pgp

**Figure 5:**
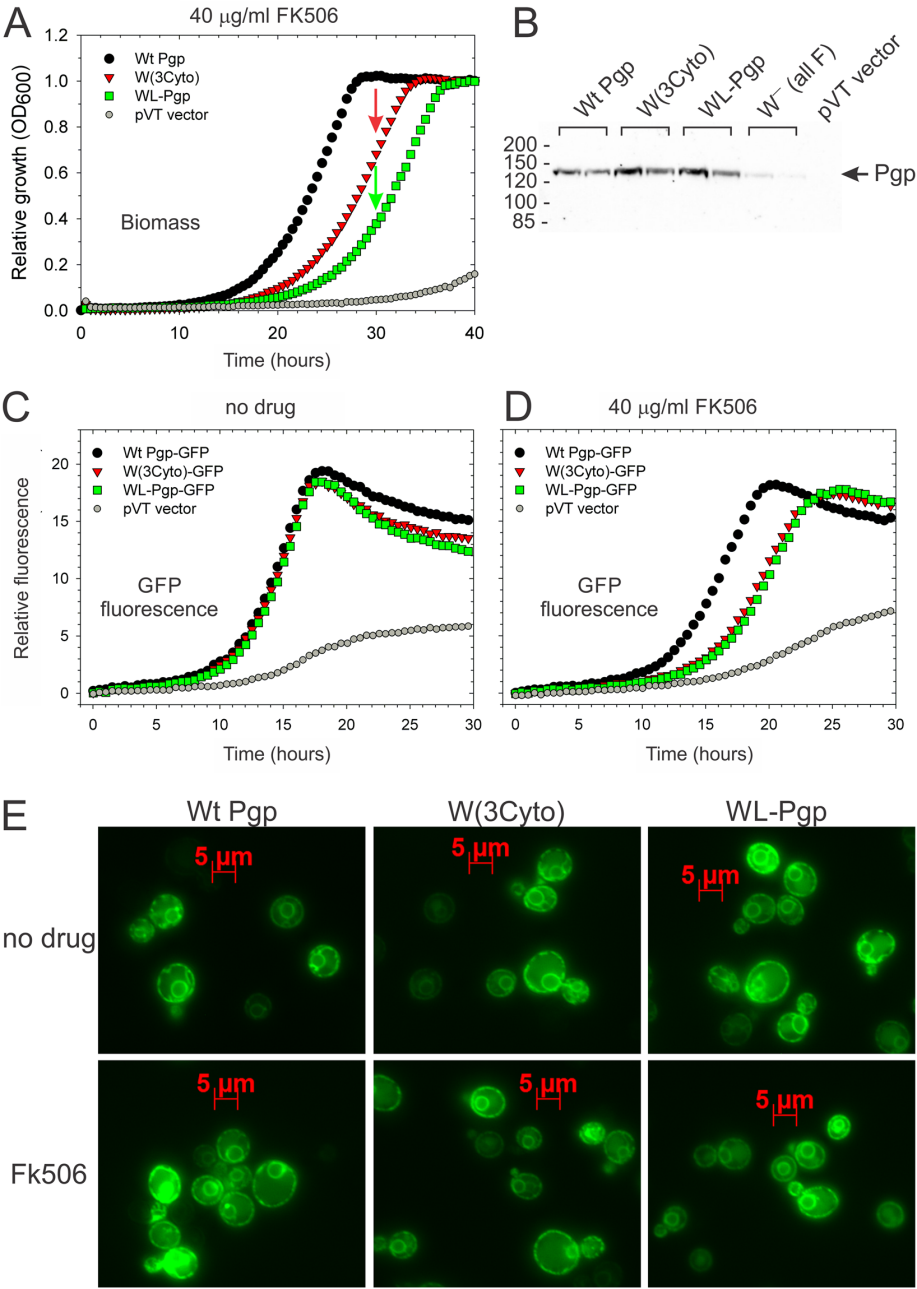
*In vivo* analyses of W(3Cyto) and WL-Pgp. (**A**) Drug resistance was continuously monitored in *S. cerevisiae* cultures expressing Pgp variants that were grown at 23 °C with occasional shaking and biomass was monitored at OD_600_. (**B**) Steady-state Pgp expression levels of each variant in microsomal membranes prepared from two mass population cultures, and assessed by Western blot using anti-Strep antibody and the enhanced chemiluminescence (ECL) detection system (Pierce). “All F” is the Trp-free mouse Pgp by Kwan *et al*.^73^ that had all 11 Trps replaced by Phe. (**C**,**D**) Drug resistance of Pgp variants containing Superfolder GFP, codon-optimized for yeast expression (see methods), was monitored in the absence or presence of FK506 under continuous shaking at 28 °C in a Biolector using 480/515 nm filters to detect GFP. E) Yeast cells expressing Pgp-GFP variants were grown in the absence (top panels) or presence of 40 µg/ml FK506, which promotes surface expression (bottom panels), to an OD_600_ of 1–2, and images were captured with a Zeiss LSM510 microscope at 60x magnification; Pgp was localized by GFP fluorescence (green) using the GFP laser and filter set. Pgp shows a bead-like surface ring typical for plasma membrane, and a smaller perinuclear ring that represents endoplasmic reticulum^83,117^.

To validate the new W(3Cyto) (containing only the three cytoplasmic Trps) and WL-Pgp we conducted a detailed comparison with Wt Pgp. In continuous FK506 drug resistance assays (Fig. 5A) with cell growth monitored every 30 min, W(3Cyto) and WL-Pgp showed delayed growth reaching ~70% and ~40% in about 30 hours, the time in which WT Pgp had reached confluency, similar to data reported in Fig. 4B. This translates to 50% growth reached in 28, 32, and 24 hours in W(3Cyto), WL-Pgp and WT, respectively. When W(3Cyto) and WL-Pgp reached confluency a few hours later, the vector control remained <20% of the Wt and Mutant biomass. Western blot analysis showed steady state expression levels of W(3Cyto) and WL-Pgp in crude microsomal membranes comparable to Wt Pgp, in contrast to “All F” Pgp that suffered from severely compromised protein expression (Fig. 5B).

To monitor protein expression in real time and localization in cells, a green fluorescence protein (GFP) was engineered at the C-terminus of Wt Pgp (Pgp-GFP). For this we tested several GFP variants, including enhanced GFP, fast-folding GFP^80^, and superfolder GFP^81,82^ (sequence is given in Supplemental Fig. S2), and found that the latter performed best in mating and drug resistance assays of Wt Pgp-GFP. Interestingly, W(3Cyto)-GFP and WL-Pgp-GFP showed delayed onset of fluorescence compared to Wt Pgp-GFP in cultures grown in the presence of FK506 (Fig. 5D), as well as the fungicide nystatin and the ionophore valinomycin (Supplemental Fig. S3). The data may suggest that delayed biosynthesis was responsible for the later onset of drug resistance in W(3Cyto)-GFP and WL-Pgp-GFP cultures. Fluorescence microscopy of W(3Cyto)-GFP and WL-Pgp-GFP cells grown in the absence or presence of FK506 showed green fluorescence at the plasma membrane as well as at the perinuclear endoplasmic reticulum that is typically seen in WT-Pgp cultures (Fig. 5E)^83^, suggesting that the mutant proteins reach their cell surface destination, where they pump out drugs and so convey drug resistance to the cells.

### Biophysical characterization of purified W(3Cyto) and WL-Pgp

**Figure 6:**
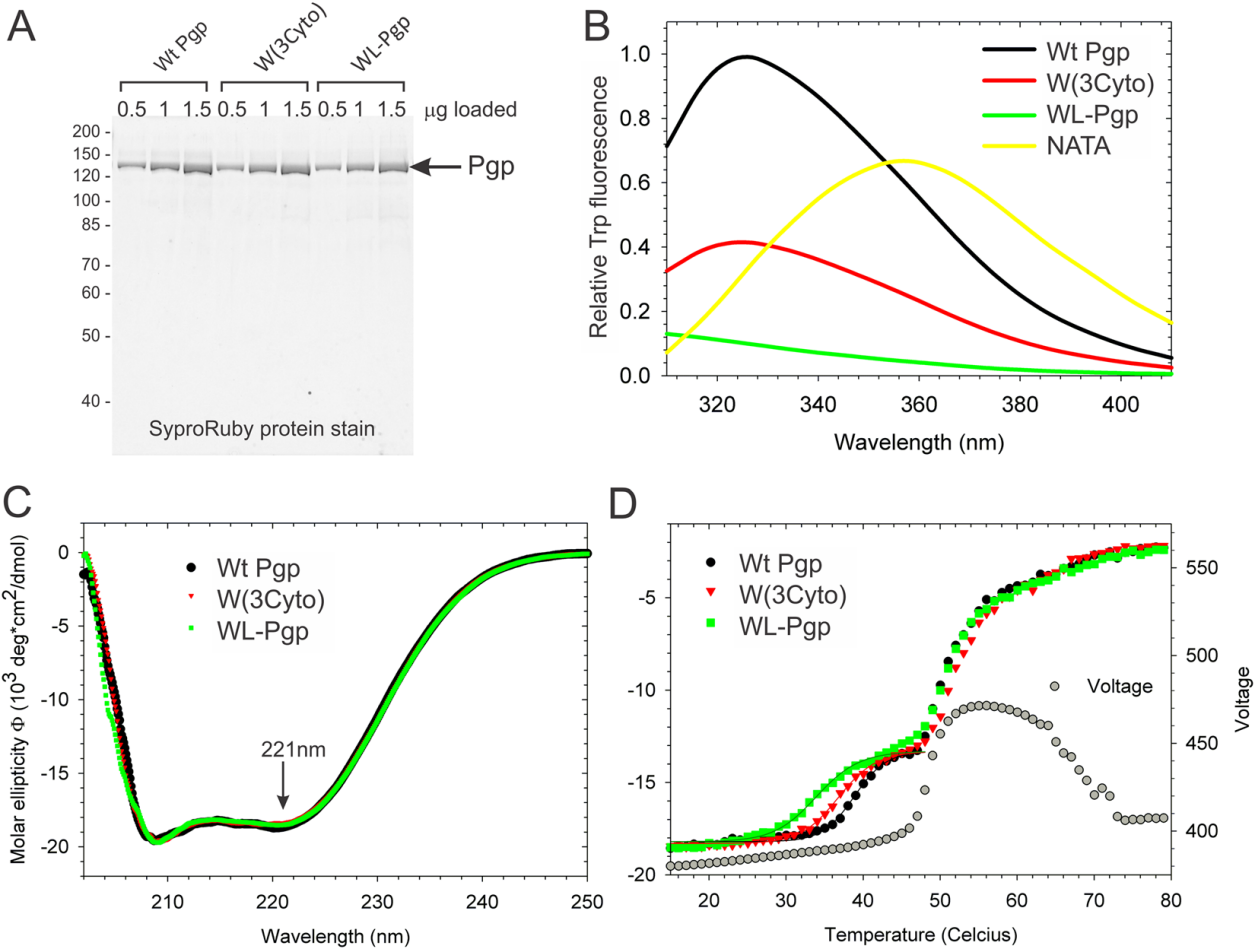
Biophysical analyses of purified W(3Cyto) and WL-Pgp. Purified proteins (0.5, 1.0 and 1.5 µg) were resolved on 10% SDS-gels and stained with the fluorescent protein stain SyproRuby. The calculated molecular size of Pgp including the TEV-cleavable twin StrepII- and His6-tags is 146 kDa; molecular weight markers are indicated in kDa on the left. (**B**) Trp fluorescence spectra of 100 nM purified WT and mutant Pgp variants recorded in 0.05% DDM buffer solution. Excitation was at 295 nm, and 1,000 nM NATA served as an internal standard. (**C**) Far-UV CD spectrum of 0.5 mg/ml Pgp variants in a 2 mm cuvette. (**D**) Thermal unfolding following the CD signal at 221 nm in the same cuvette, scan rate was 1 degree/min. The voltage applied on the photomultipliers is shown in gray circles (right axis).

Trp-reduced W(3Cyto) and WL-Pgp proteins can be purified from *S. cerevisiae* fermentor cultures using His_6_ and dual StrepII-tags with excellent yield and purity (see methods)^47,79^. A single Pgp protein band was apparent on SDS-PAGE gels and no contaminating proteins were detected even with the highly sensitive fluorescent stain SyproRuby affirming that the double-affinity purified proteins were very pure (>95%, Fig. 6A). The yield of WT Pgp (~13 mg/200 g *S. cerevisiae* cells) was a little lower than previously reported (20 mg/200 g cells)^47,84^, as expected due to the additional affinity purification step. The yields of W(3Cyto) and WL-Pgp (~10 mg/200 g cells and 6–8 mg/200 g cells, respectively) were somewhat lower than for WT. As expected, the fluorescence emission intensity diminished from Wt Pgp to W(3Cyto) to WL-Pgp (Fig. 6B). The fluorescence emission maximum (λmax) of 326 nm was the same for Wt and W(3Cyto) Pgp (Fig. 6B); this is consistent with an, on average, hydrophobic environment of the detectable Trps. No Trp fluorescence peak was detectable for WL-Pgp, attesting to the purity of the preparations, as most contaminating proteins would be expected to contain Trp.

The far-UV CD spectrum of detergent-solubilized Pgp exhibited minima at 208 and 222 nm, as previously reported, and is typical for an α-helical protein (Fig. 6C)^84,85^. The shape of the far-UV CD spectra of W(3Cyto) and WL-Pgp was indistinguishable from Wt samples, suggesting that secondary and, in all likelihood, tertiary structure was maintained in the Trp mutants. Two thermal unfolding transitions were observed by CD (Fig. 6D). The first one was accompanied by a loss in ellipticity of 32 ± 2%, with a Tm of 38.0 ± 0.5 °C seen in Wt-Pgp. A second transition followed immediately after a sharp increase in the PMT voltage profile (gray circles, right axis). The increase in voltage suggested sample aggregation^85^, and coincided with a visible precipitation in the sample cuvette after heating above 50 °C. A comparison of the thermal unfolding profiles showed that the first transition was shifted to slightly lower temperatures in the W(3Cyto) (36.8 ± 0.5 °C), and significantly lower in the WL-Pgp (33.5 ± 0.3 °C). The second transition, assigned to protein aggregation, resulted in a further loss in ellipticity of 68% and was the same for all three Pgp variants. The data suggest that removal of the three cytoplasmic Trps, W158, W799 and W1104, caused some thermal destabilization (>3 degrees) of the NBDs, which may also be reflected in the lower protein yields obtained for the WL-Pgp.

### ATPase activity

**Figure 7:**
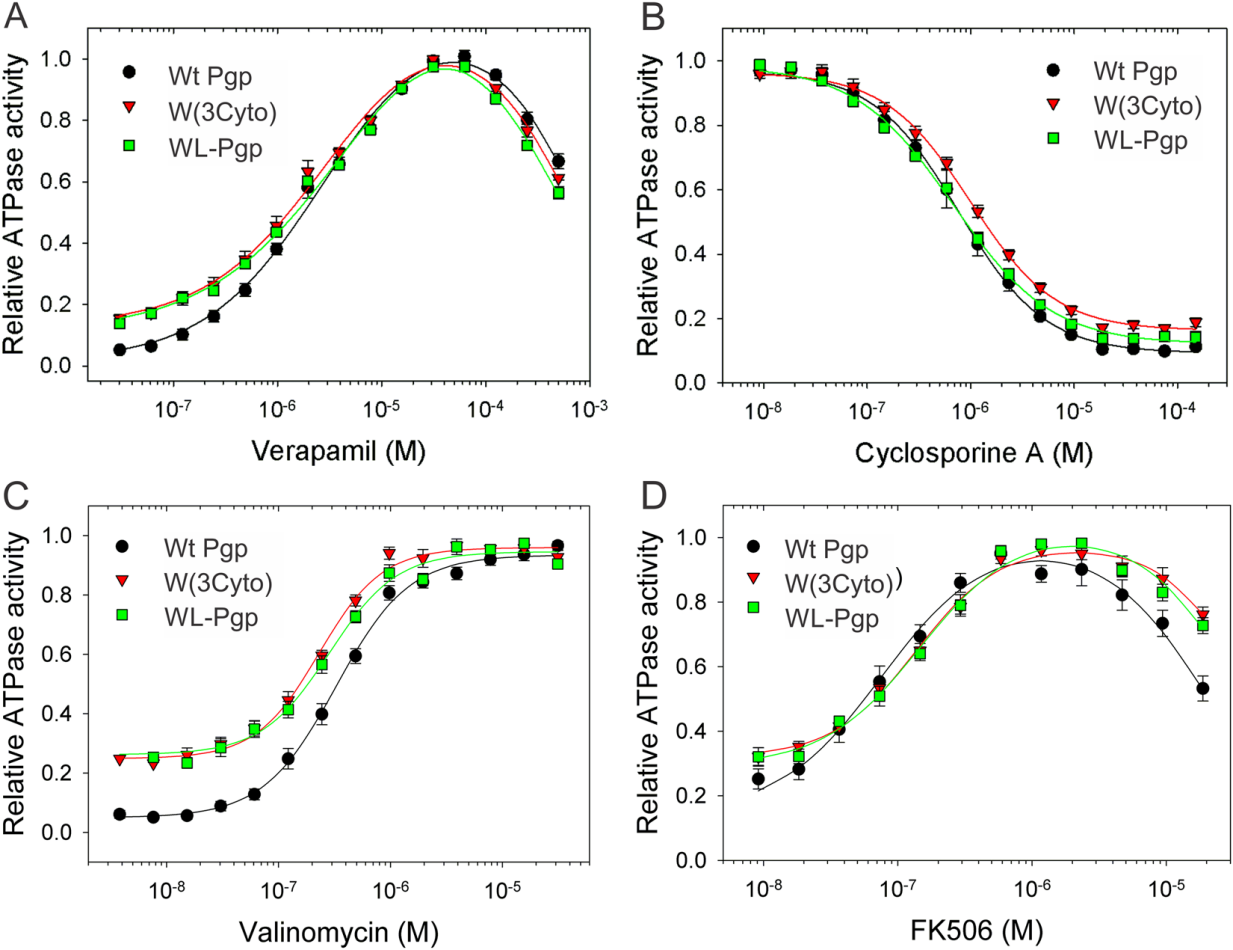
Drug stimulation of ATPase activity of purified W(3Cyto) and WL-Pgp. (**A**) ATPase activity of purified proteins was measured with increasing verapamil concentrations, or (**B**) with increasing cyclosporin A concentrations in the presence of 30 μM verapamil. (**C**) Dose-response curves with increasing concentrations of valinomycin, or (**D**) with FK506. Symbols represent the mean of 4 independent experiments ± SEM. Data were fitted using a modified Hill equation (lines).

The kinetic parameters of MgATP hydrolysis of the purified proteins were determined in the presence of *E. coli* polar lipid, after activation with DTT (to reduce disulfide bonds)^86,87^ and are given in Table 2. The specific verapamil-stimulated ATPase activity (Vmax) was high at around 6 μmol/min/mg, and was similar for all three Pgp variants, assuring that removal of the eight TM Trps or all eleven Trps did not affect the hydrolysis rates. The Km(MgATP) of WT and WL-Pgp were in the mM range as previously reported^47,84^, and were somewhat higher than in W(3Cyto) (p < 0.05). The most notable differences were seen in the ‘basal’ ATPase activity of W(3Cyto) and WL-Pgp in the absence or at low verapamil, valinomycin and FK506 concentrations that were significantly higher than for WT Pgp (p < 0.05; Table 2, Fig. 7A,C). This resulted in a lower degree of stimulation observed for the mutants. For example, the degree of stimulation by verapamil in wild-type protein was 26.7-fold (5.6/0.21 μmol/min/mg), which was reduced to 6.1-fold (6.3/1.03 μmol/min/mg) and 7.9-fold (5.5/0/7 μmol/min/mg) for W(3cyto) and WL-Pgp, respectively (Table 2). The higher basal activity may suggest that, in the absence of drug, ATP hydrolysis in the NBDs is more uncoupled from the TMDs in the W(3Cyto) and WL-Pgp variants. Increased basal ATPase activity has also been observed for Pgp when either the cytoplasmic sites of the TMDs, the ICL, or the NBDs were linked closely together (Loo *et al*., Verhalen and Wilkens)^88–91^. It is possible that restricting the motion of the NBDs results in a greater probability of generating the ATP-bound sandwich configuration, which in turn may explain the increase in basal activity^91^.

**Table 2:** ATPase activity.

	WT Pgp	W(3Cyto)	p	WL-Pgp	p
Specific verapamil-stimulated ATPase activity (μmol/min/mg)^a^	5.6 ± 1.0 (7)^b^	6.3 ± 1.1 (7)	0.28	5.5 ± 0.71 (7)	0.83
K_m_(MgATP)^a^	0.75 ± 0.20 (5)	0.40 ± 0.07 (5)	0.006	0.65 ± 0.07 (5)	0.32
Basal ATPase activity (μmol/min/mg)	0.21 ± 0.15 (6)	1.03 ± 0.48 (6)	0.002	0.70 ± 0.26 (6)	0.011
Verapamil EC_50_ (μM)^c^	2.0 ± 0.36 (5)	2.0 ± 0.67 (5)	0.10	2.5 ± 0.17 (5)	0.043
Valinomycin EC50 (μM)	0.33 ± 0.08 (8)	0.23 ± 0.03 (6)	0.02	0.34 ± 0.09 (6)	0.84
FK506 EC_50_ (μM)	0.084 ± 0.03 (6)	0.14 ± 0.03 (5)	0.009	0.16 ± 0.04 (5)	0.004
Cyclosporin A IC_50_ (μM)^d^	0.81 ± 0.37 (5)	0.99 ± 0.20 (5)	0.35	0.67 ± 0.04 (5)	0.42

^a^Vmax and Km were determined in the presence of 30 μM verapamil to maximally stimulate ATP hydrolysis using simple Michaelis-Menten kinetics with the SigmaPlot Software package; no cooperativity for MgATP hydrolysis was observed.

^b^Means ± standard deviations are given with the number of independent experiments (n) indicated in brackets; p-values were calculated using the Paired Student’s T-Test.

^c^The concentration required for 50% stimulation of the ATPase activity (EC_50_) was calculated from individual fits of which the average ± SD are shown in Fig. 7A,C, and D.

^d^Inhibition of the verapamil-stimulated ATPase activity (IC_50_) by Cyclosporin A was determined from individual fits of which the average ± SD are shown in Fig. 7B.

Importantly, the affinity for these substrates that stimulate the ATPase function in a concentration-dependent manner was very similar to Wt Pgp (Fig. 7) with 50% stimulation (EC_50_) observed at around 2–2.5 μM verapamil and ~0.3 μM valinomycin, while small but significant differences were seen with FK506 (0.14 and 0.16 μM in W(3CYTO) and WL-Pgp vs. 0.084 μM in WT Pgp, respectively). Biphasic behavior of ATPase stimulation at low concentration and ‘back”-inhibition at higher concentrations is typically seen with verapamil and FK506 (Fig. 7A,D). Also, the concentration required for 50% inhibition (IC_50_) of the verapamil-stimulated ATPase by the inhibitor cyclosporine A was very similar in WT, W(3Cyto) and WL-Pgp (Fig. 7B, Table 2). In summary, the data indicate that the functional properties, polyspecificity and drug binding affinities of the WT protein were preserved in the new W(3Cyto) and WL-Pgp variants. Thus, we created excellent new tools to distinguish changes in the cytoplasmic versus TMDs of Pgp. The new WL-Pgp, or W(3Cyto), can be used as background to introduce Trps in strategic positions.

## Discussion

The goal of this study was to generate a Trp-free Pgp (WL-Pgp) which can then serve as background for the strategic re-insertion of Trps to study drug binding, nucleotide interactions, and the interplay between both. Preliminary data had indicated that a conservative replacement was not optimal for all 11 Trps^73,74^. Thus, we used directed evolution to identify the best substitution in each position and obtained an intermediate construct where just the eight Trps in the TMDs were removed, W(3Cyto), and WL-Pgp. Both constructs had wild-type-like drug-stimulated ATP hydrolysis rates, and similar binding affinities for substrates and inhibitors, with less than 2-fold difference seen for verapamil-, valinomycin- and FK506-induced stimulation of ATPase activity, and no significant difference observed for inhibition by cyclosporine A. Wild-type-like CD spectra indicate no change in secondary structure. Three (possibly related) functional parameters were affected, as Trp removal resulted in a slight loss of stability, as seen from the decreasing melting temperature, in slightly reduced growth rates, and in increased basal ATPase activity (in the absence of drug). As expected, W(3Cyto) Pgp showed a significantly reduced Trp fluorescence signal, WL-Pgp none at all.

With existing knowledge, some of the optimal replacement amino acids found by directed evolution would not have been predicted, although in retrospect all can be explained to a certain degree. Analysis of Pgp homologs could have given useful clues for suitable replacements in some, but not in all cases (Table 3, Supplemental Table S1). Especially for the Trp residues in the membrane portion, the most frequent substitutions seem specific for location relative to the membrane and/or for the local secondary structure environment, and are often biased towards non-conserved amino acids. Substitution matrices developed for soluble proteins^92,93^ appear to be less reliable for transmembrane regions. The eight Trp residues in the membrane portion of Pgp are all located at the level of the interface between the hydrophobic core of the bilayer and the bulk water; all except W228 are located toward the outside of the protein. A preferred position for Trp residues at the end of the membrane portion of a transmembrane helix has been described before^94–97^. Among the amino acids that share this preference are Tyr, Pro, and His^94–96,98^. Trp, Tyr and Pro are slightly more likely to be found on the extra-cytoplasmic outer side than on the cytoplasmic inner one, specifically in extra-cytoplasmic loops^94,96,99^, which explains several of the substitutions found for the outer leaflet residues W208, W311, and W851 (Fig. 2, Table 3). Some Pgp homologs actually have a Pro in the position equivalent to W208 or W851 (Supplemental Table S1). Pro was also observed as replacement for W694 in the inner leaflet, as was His as replacement for W44 and W132. In addition, Arg and Lys are indeed found frequently at the end of a transmembrane helix segment (see W208), although with a strong bias for the cytoplasmic side (W44). Arg and Lys have been found to “snorkel”; while their positively charged side chain interacts with the negative charges of the phospholipid headgroups, the C_α_ atom can be located deeper in the hydrocarbon portion of the membrane^100^. Trp can show the same tendency, although to a lesser extent^100^. In proteins in general, i.e. not restricted to membrane proteins, among the amino acids frequently found in turns are Pro, Gly, and Ser^101^, as seen here for W208 (Pro), W851 (Pro), W694 (Pro, Ser), W704 (Gly), and W1104 (Gly) (Table 3). W694 is the least conserved of all Trp residues in Pgp, in agreement with its position at the end of the linker region connecting both halves of Pgp where the variability is high.

**Table 3:** Substitution Patterns of the 11 Trp Residues in Pgp.

Trp residue	Directed Evolution	Analysis of Pgp Homologs^a^		Location
	Preferred Substitutions	Conservation	Preferred Substitutions	
**Outer Trp block**				
W208	R P^b^ F K V	very strong	A P	outer leaflet; in loop between TM helices 3 and 4
W228	A L S T M G	weak	I Y M E F Q	inner vestibule; close to drug binding site(s)
W311	F Y	strong	R	outer leaflet; in TM helix, two turns from C-terminus
W851	P	very strong	P Y	outer leaflet; in loop between TM helices 9 and 10
**Inner Trp block**				
W44	R V H A	moderate	S C G	inner leaflet; at N-terminus of TM helix
W132	I L H	fairly strong	F	inner leaflet; at end of TM segment of long helix
W694	P L T S I	very weak	L F T R	inner leaflet; at N-terminus of elbow helix
W704	G L V Y F	strong	I C V L	inner leaflet; in turn between elbow helix and TM helix
**Cytoplasmic Trps**				
W158	Y	very strong	F Y	in short helix coupling intracellular loop to NBD
W799	F Y	strong	F Y	in short helix coupling intracellular loop to NBD
W1104	G Y V	fairly strong	F H	in short helix in NBD2

^a^Data are based on a BLAST search of the 250 (or 1000) closest relatives to mouse Pgp (see Supplemental Table S1)^118^.

^b^Replacements found in W(3Cyto) and WL Pgp are underlined.

Of the eight Trp residues in the TM region, W228 is in a special position as it lines part of the inner vestibule which hosts the drug translocation pathway(s)^55^. The wide variety of amino acids observed in this position (Fig. 2, Table 3) might reflect the broad specificity of Pgp with regard to the transport substrate.

In soluble proteins, conservative Trp replacements are generally well tolerated^92,93^. This also applies here for the cytoplasmic Trps W158, W799, and W1104. However, the strictness of the preference for Tyr in position W158 and for Phe and Tyr in position W799 was surprising (while a few other replacements were found, these showed very little activity; see Fig. 4). W158 and W799 are located in equivalent positions in either half of Pgp at the apex of the intracellular loop in a short helix that couples the ICL to the NBD. Mutations disrupting the transmission interface between ICL1/NBD1 and/or ICL3/NBD2 have been reported to cause misfolding of human Pgp that may be rescued by molecular chaperones^102,103^. Thus, W158 and W799 may have unique functions in maintaining structural integrity of the connecting loops and/or interactions with the NBD^74,79,102,103^ that is best preserved by conservative Tyr substitutions.

In conclusion, our results show that in membrane proteins no single amino acid is a universally ideal substitute for Trp. Within a membrane protein, amino acids can exist in multiple environments including the water soluble intracellular or extracellular regions of the protein, the hydrophobic core of the membrane bound protein domains, or the membrane water interface. In certain local environments, substitution by a non-conserved amino acid may in fact be favorable under a specific condition. In the future, studies using the directed evolution methods developed for this project should be able to help to produce membrane protein specific substitution matrices that can specifically account for local environments that can bias replacement of a specific amino acid.

## Materials and Methods

### Materials

FK506, cyclosporine A, doxorubicin, and valinomycin were purchased from A.G. Scientific (San Diego, CA). Fluconazole was from LKT Laboratories (Saint Paul, MN). Verapamil, and nystatin were from Sigma Aldrich (Saint Louis, MO). 5-FOA and G418 were from US Biological (Swampscott, Massachusetts). E. coli lipids (Polar Extract) was purchased from Avanti (Alabaster, AL), n-dodecyl-β-D-maltopyranoside (DDM) was from Inalco (Italy).

### Construction of mutant libraries by recombinant PCR

The starting template for this study was codon-optimized mouse *mdr1a* Pgp (*opti-mdr3*, *abcb1a*, GenBank JF834158), herein referred to as Wt Pgp, in the pVT expression vector (pVT*mdr3*)^84,104^. Our strategy for site-saturation mutagenesis was to allow any amino acid other than Trp (TGG) by using a mixture of three oligonucleotide primers (“Trp^−^”): VNN (does not code for Cys, Phe or Tyr), NHN (does not code for Arg, Cys or Gly), or NNH (does not code for Met). Alternatively, fully degenerate primers (“Trp^+^”) replaced the Trp codon with NNN, encoding all 20 amino acids including Trp. The Trps from each block were replaced by an overlap/extension recombinant PCR method using Phusion polymerase (Thermofisher) and the site-saturation mutagenic oligonucleotides containing the Trp^+^ or Trp^−^ degenerate codons at each of the Trp positions, see Supplemental Fig. S1. The resulting PCR products and linearized vector (pVTmdr3 digested to remove DNA segment being replaced with PCR product) were ethanol precipitated before being co-transformed by lithium acetate into *S. cerevisiae*, strain JPY201 (MAT**a** ura3 Δste6::HIS3 gal2 lys2-801 leu2–3,2–112 trp1-1(am) ura3-52)^77,105^, for reintroduction of the mutant block into pVTmdr3 by homologous recombination. Cells from each transformation sample were divided onto ten plates of uracil deficient medium and incubated at 30 °C until colonies formed. Colonies were then counted to estimate the total number of transformants obtained for a mutant block. 15–20 separate transformation samples were produced and plated at a time, and 2 to 3 independent sets of transformation samples were produced for each mutant block.

### ‘Outer Trp’ mutant selection and identification

Mating was used as a first tier screening tool because it is a stringent test of mutant function as we previously demonstrated^74,79^, and because it gives single colonies of functional mutants. For initial mutant selection, Pgp expressing JPY201 cells were mated with an α-type yeast strain DC17-U (MAT**α** his1 Δura3::KANMX) to test for complementation of the *Ste6* yeast pheromone transporter by Pgp^75,77,105^. DC17-U was generated for this study from DC17^77^ by replacing a segment of the *Ura3* gene with the KANMX segment using the M3927 ‘marker swap’ plasmid, selecting for transformants on YPD medium containing 400 µg/ml G418 (Genticin), and selecting for auxotrophic cells with uracil medium containing 1 mg/ml 5-Fluroorotic acid (5-FOA), allowing uracil to still be used as the selection for plasmid containing cells after mating^106,107^. The mating assay was adapted for effective selection of thousands of colonies as follows and higher throughput of samples: Using a replica plating tool, colonies from uracil-deficient plates were transferred to YPD plates containing a lawn of DC17-U tester strain cells (4 × 10^7^ cells/ml), incubated for 6–8 hours at 30 °C, before being replica plated on minimal medium to identify the colonies that had successfully mated. Colonies that formed on minimal media plates were transferred to 48 well plates containing uracil deficient media as a reservoir for storage and further testing. For the ‘outer Trp block’ (Supplemental Fig. S1A), 300,000 transformants yielded a total of 1,116 single colonies that successfully mated, i.e. were able to grow to decent size colonies on minimal medium (Table 1).

As a second, independent screen we tested drug resistance against FK506 and doxorubicin. Aliquots of the 1,116 colony stocks were grown to late-log phase in 96 well plates in uracil-selective media containing 50 µg/ml FK506 or 30 µM doxorubicin, and the A_600_ determined relative to vector control cells (transformed with empty pVT vector), and wild-type *pVTopti-mdr3* strains as described^74,79^. 624 samples exhibited drug resistance of at least 50% of Wt Pgp; from these DNA was recovered by colony PCR, and the PCR amplified DNA were sequenced to identify the mutations at each Trp position (Beckman Coulter Genomics, Danvers, MA). Sequencing of multiple single colonies from successfully mating samples revealed that the populations were generally homogeneous for a single mutant, but occasionally mating samples contained up to four distinct mutant plasmids. Trp^+^ libraries yielded more transformants that successfully mated but more than half of them carried a Trp in at least one of the four positions. At this stage, a total of 316 unique mutants were recovered that did not contain Trp in any of the 4 mutated sites; 65 of which derived from completely degenerate “Trp^+^” primers, and 251 (79.4%) from the “Trp^−^” primers.

### ‘Inner Trp’ mutant selection and identification

For the four ‘Inner Trp’ mutations, plasmid DNA from yeast colony stocks of the 316 unique Trp-free mutants were extracted using a variation of the Promega Wizard® miniprep kit and the *mdr3* open reading frame PCR amplified. PCR amplified DNA was obtained from 272 of the 316 unique mutants, and those were divided into 12 pools to serve as template for recombinant PCR using either mutagenic “Trp^+^” or “Trp^−^” primers (Supplemental Fig. S1B). Assembly of PCR products containing the mutant libraries, re-introduction into the pVT*mdr3* plasmid by co-transformation with linearized pVT*mdr3* vector into *S. cerevisiae* strain JPY201, mating and drug selection were all performed as described for the ‘outer Trp’ block above.

For the ‘inner Trp’ block, 200,000 transformants yielded a total of 1,143 single colonies that successfully mated. DNA was recovered by colony PCR^108^ from 768 samples, and PCR products sequenced to eliminate samples that contained a Trp codon at any of the four inner Trp positions W132, W208, W694 and W704. Subsequently, 196 DNA plasmids were extracted from yeast, propagated in *E. coli*, and purified plasmids retransformed into naïve JPY201 yeast. Cultures of the transformants were screened again for mating efficiency. As a second screen, drug resistance against 30 µM doxorubicin was assayed in 96 well plates as above to select the most active samples. The purified plasmid DNA from the most active 151 samples were re-sequenced. At this stage, 102 plasmids (72 unique mutants) were confirmed Trp-free at any of the four outer Trp and the four inner Trp sites. DNA from the 72 unique mutants was again re-transformed into JPY201 yeast cells for more detailed *in vivo* functional analysis of fungicidal drug resistance and yeast mating efficiency, which were performed essentially as previously described^74,79,84^. The mutant W44H/W132L/W208P/W228M/W311Y/W694I/W704I/W851P was identified from this pool for its high activity in both assays, renamed W(3Cyto) for short, and retained for further studies and construction of a fully Trp-free Pgp.

### Cytoplasmic Trp mutant selection and identification

pVT-mdr3 containing the W(3Cyto) mutations served as template for substitution of W158, W799, and W1104 using “Trp^+^” or “Trp^−^“ mutagenic primers in overlap extension PCR. Assembly of the mutants, reintroduction of the mutants into the plasmid, and screening for mating activity were performed as described above for the ‘Outer’ and ‘Inner’ Trp mutants. Approximately 500,000 transformants yielded 346 colonies after mating with DC17-U. Colony PCR and sequencing identified 265 mutants that were Trp free at W158 and W799. The plasmid DNA from these yeast was extracted, propagated, and re-transformed into naïve JPY201 cells. Successive mating assays with the re-transformed mutant library narrowed the pool to 15 mutants with high mating activity. These mutants were again extracted, full length sequenced, and re-transformed into naïve yeast cells for detailed analysis, as described for the ‘Inner Trp’ mutants.

### *In vivo* drug resistance and cellular localization

To allow for *in-vivo* expression and localization analyses, a green fluorescence protein (GFP) was fused to the C-terminus of the WT Pgp gene in the pVTmdr3 vector. Briefly, a cassette containing, in order, a tobacco etch virus (TEV) protease site, a “superfolder” GFP, two StrepII tags and a His6 tag was codon-optimized for expression in *S. cerevisiae* and *P. pastoris* yeast, synthesized by GeneArt/ThermoFisher and subcloned via existing 5′-*XhoI* and 3′-*AgeI* sites. The cassette sequence and a more detailed description are given in Supplemental Fig. S2. The ORF of the entire pVTmdr3-TEV-superfolder GFP-2xStrep-His6 construct was confirmed by DNA sequencing, deposited in GenBank (accession Number MN384969), and hereafter referred to as Pgp-GFP. The entire GFP cassette was then subcloned into W(3Cyto) and WL-Pgp via 5′-*XhoI* and 3′-*AgeI* sites to generate W(3Cyto)-GFP and WL-Pgp-GFP. Routine plasmid manipulations and preparations were performed in XL10-Gold (Agilent) *E. coli* cells throughout this study.

Plasmids were transformed into JPY201 yeast by the Li-acetate method^109^. Continuous growth assays were performed in 48-well “flower” plates in a BioLector microbioreactor (m2p-labs) at 28 °C, 1,000 rpm with airflow and humidity control monitoring biomass production (A_600_) and GFP fluorescence with appropriate excitation/emission filters (488 nm/>510 nm). The trend of relative growth of WT > W(3Cyto) > WL-Pgp in a simple 96 well plate reader (BioRad) at room temperature was very similar to the trend of their GFP-tagged counterparts Pgp-GFP > W(3Cyto)-GFP > WL-Pgp-GFP in a BioLector (compare Fig. 6A,D), albeit cells generally grew faster in the controlled conditions of the BioLector.

### Protein purification and ATPase assays

To facilitate protein purification, an expression cassette coding for TEV-cleavable twin StrepII tags and a His_6_ tag was engineered at the C-terminus of mdr3 that translates to LEENLYFQGGGASGGSWSHPQFEKAAAGGGSGGGSWSHPQFEKGSGHHHHHH*TG to generate WT-Strep_2_-His_6_, W(3Cyto)-Strep_2_-His_6_ and WL-Pgp-Strep_2_-His_6_. The sequence is identical to Supplemental Fig. S2 but devoid of GFP that was excised by its flanking *NarI* sites. The plasmids were named pVT-opti-mdr3-Strep_2_-His_6_, pVT-W(3Cyto)-Strep_2_-His_6,_ pVT-WL-opti-mdr3-Strep_2_-His_6_. For purification from *S. cerevisiae*, plasmids were transformed into the BY4743 strain (MATa/α his3Δ1/his3Δ1 leu2Δ0/leu2Δ0 LYS2Δ0lys2Δ0 met15Δ0/MET15 ura3Δ0/ura3Δ0)^110^ that grows to higher densities than JPY201. Fourteen-liter cultures were grown in a New Brunswick BioFlow IV fermentor to a maximal A_600_ of 5 to 6, which produced 200–250 g of cells. Microsomal membrane preparation, Pgp solubilization in 0.6% DDM in buffers containing 500 mM NaCl, and purification based on the affinity of the Pgp His_6_ tag for Ni-NTA were performed as described^47,84,111^. Pgp proteins were subjected to an additional purification step on StrepTactin superflow resin (Qiagen, Valencia, CA) in Buffer A (50 mM Tris pH 8, 10% glycerol, 500 mM NaCl, and 0.05% DDM) with 1 mM DTT, and eluted by competition with 4 mM desthiobiotin^47^.

The concentrations of purified Pgps were initially determined from the absorbance at A_280_ nm using a calculated molar extinction coefficient Ɛ including the purification tags of 126,630 for the WT^85^, 84,620 for W(3Cyto), and 72,5900 for WL-Pgp, respectively. Protein concentrations were verified by the bicinchoninic acid (BCA) protein assay using BSA as a standard. Finally, increasing protein amounts of WT and Trp mutants were resolved side-by-side on SDS/PAGE gels, stained with Sypro Ruby followed by Coomassie Brilliant Blue, and Pgp was quantified using ImageJ (http://rsbweb.nih.gov) to compare mutant Pgp levels with the two dyes^85^. Sypro Ruby, which interacts strongest with Lys, Arg, and His residues and less strongly with Tyr and Trp residues, gave more consistent staining of W(3Cyto) or WL-Pgp variants.

### Functional and biophysical analyses of purified Trp-variants

To determine the ATPase activity, purified Pgp (0.5–1.0 µg) in detergent was activated by incubation with 10 mM DTT and 1% (w/v) *E. coli* polar lipids for 15 min at room temperature^86,87^. The rate of ATP hydrolysis was measured at 37 °C, by an ATPase-linked enzyme assay^87^, in the absence and presence of drugs, and statistical analysis were done as described^74,79^. For biophysical studies, Pgp was exchanged into SEC buffer (20 mM HEPES pH 7.4, 150 mM NaCl, 10% glycerol, 0.05% DDM,) by size exclusion chromatography (SEC) on Superdex 200 (1 × 30 cm column)^85^. For fluorescence studies, the 2X Strep II tag was removed by incubating a protein aliquot on ice with TEV protease at a Pgp:TEV ratio of 10:1 (w/w) for 2 hours prior to size exclusion chromatography. Pgp intrinsic Trp fluorescence emission was measured of proteins diluted to 100 nM and the steady state fluorescence emission scanned from 310 to 410 nm with an excitation wavelength of 295 nm in a Fluorolog-3 spectrofluorometer (Horiba) at room temperature. Emission spectra were corrected by comparing the technical spectra of tyrosine and tryptophan with the corrected spectra^112^.

Circular Dichroism (CD) spectra were recorded at 15 °C at a protein concentration of 0.25–0.55 mg/ml in a 2 mm cuvette using a thermostated CD spectrophotometer from Jasco J-815 with the Spectra ManagerTM II software for control and data analysis. Reference and sample buffers contained SEC buffer with 0.1 mM TCEP. Protein unfolding was monitored at 221 nm at a heating rate of 2 K/min. CD protein unfolding data were analyzed between 15 °C and 48 °C (before protein aggregation occurred); solid lines in Fig. 6D are fits using the Sigmaplot software to the data points (symbols) using a two-state transition of a monomer from a folded to unfolded state as described by Greenfield to obtain the unfolding temperature (Tm)^113^.

## Supplementary information

Supplementary Information.
