# Supplementary material for: Replacing the eleven native tryptophans by directed evolution produces an active P-glycoprotein with site-specific, non-conservative substitutions
Source: Sci Rep. 2020 Feb 21;10:3224. doi: 10.1038/s41598-020-59802-w (PMC7035247; doi:10.1038/s41598-020-59802-w)
Supplement: Supplementary file 1 — Supplementary Information. [file 41598_2020_59802_MOESM1_ESM.pdf]

Supplementary information

Replacing the eleven native tryptophans by directed evolution produces an active P-glycoprotein with site-specific, non-conservative substitutions

Douglas J. Swartz[1,2#], Anukriti Singh[1], Narong Sok[1], Joshua N. Thomas[1], Joachim Weber[2,3*] and Ina L. Urbatsch[1,2*]

[1]From the Department of Cell Biology and Biochemistry, and [2]Center for Membrane Protein Research, Texas Tech University Health Sciences Center, Lubbock, Texas, USA

[3]Department of Chemistry and Biochemistry, Texas Tech University, Lubbock, Texas, USA

*) To whom correspondence may be addressed: ina.urbatsch@ttuhsc.edu and joachim.weber@ttu.edu

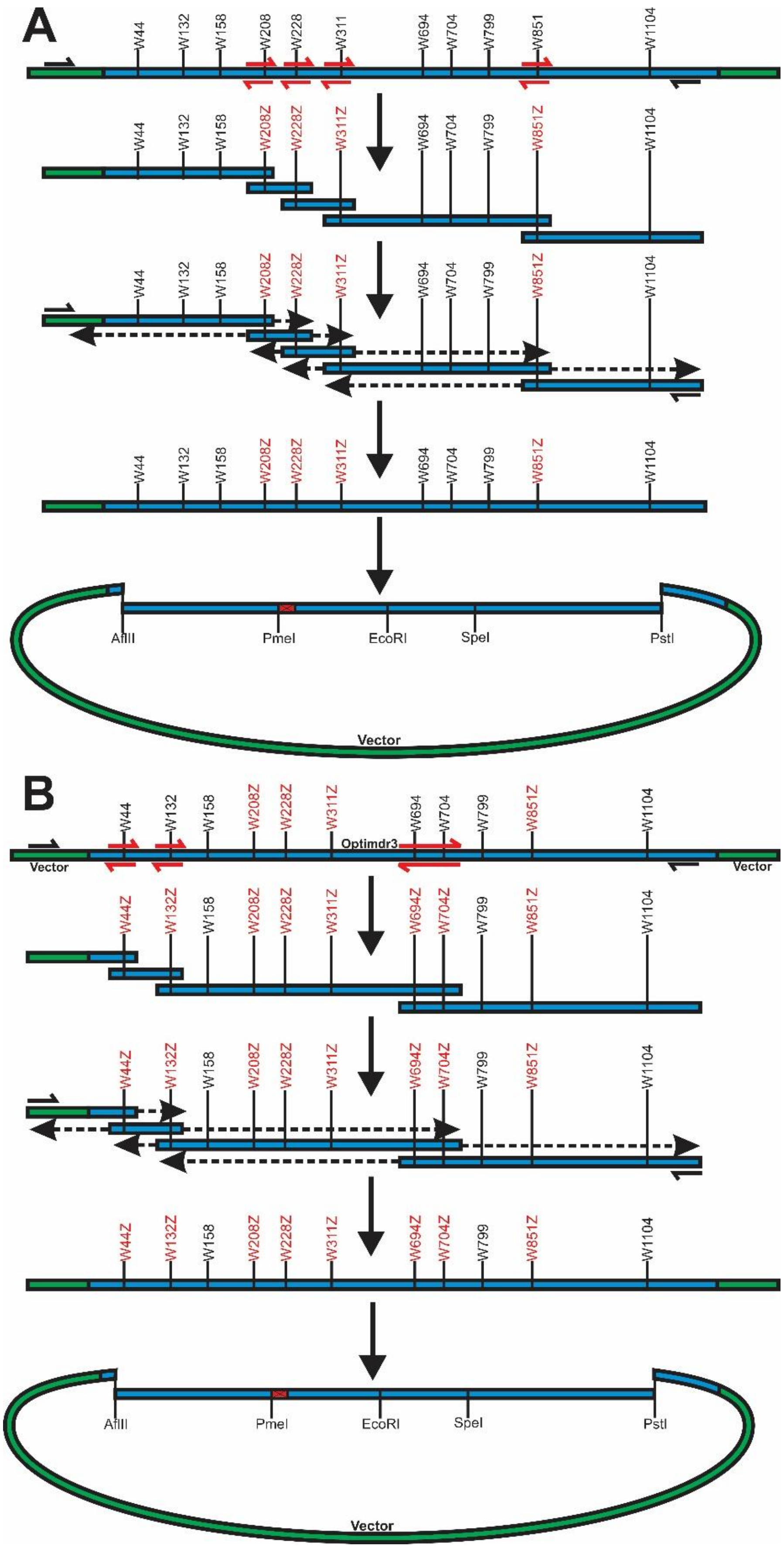
A
W44
W132
W158
W208
W228
W311
W694
W704
W799
W851
W1104
W208Z
W228Z
W311Z
W851Z
AflII
PmeI
EcoRI
SpeI
PstI
Vector
B
Optimdr3
Vector
W44Z
W132Z
W694Z
W704Z

Supplemental Figure S1: Strategy for construction of the Trp mutant blocks by site-saturation mutagenesis. An overlap extension PCR approach was used to generate a mutant library. First, overlapping mutant fragments were generated through a standard PCR protocol with mutagenic primers at each Trp position (red arrows) and non-mutagenic flanking primers (black arrows), using Phusion Hot Start II polymerase (Thermofisher) with pVT-*mdr3* as template. Either mutagenic "Trp^{-}" primers replaced the Trp codon with a mixture of degenerate codons (VNN, NHN and NNH) that allow expression of the 19 remaining amino acids (Z), or mutagenic "Trp^{+}" primers with fully degenerate codons (NNN) that allow expression of all 20 amino acids, respectively. These fragments were gel purified, quantified, and used in equal ratios for the second "anneal and fill" PCR reaction, which contained no additional PCR primers, to extend the fragments from the overlapping sequences. Then, flanking primers were used to amplify the full length Trp mutant blocks in a final PCR reaction. The flanking primers extended the mutant blocks ~100 bp beyond the respective restriction sites for re-introduction into pVT-*mdr3* plasmid by homologous recombination in *S. cerevisiae* [1]. For recombination, pVT*mdr3* was digested with *AflII* and *PstI* restriction enzymes, gel purified, mixed with a two-fold excess of the Trp mutant PCR library and co-transformed into *S. cerevisiae*, strain JPY201(MATa ura3 Δste6::HIS3) [2,3]. The pVT*mdr3* plasmid used for recombination was modified by adding an in-frame Stop codon and *PmeI* restriction site to the portions of mdr1a replaced by the Trp mutant blocks to prevent contamination from undigested plasmid DNA.
A) outer interface Trp mutant block, and B) inner interface Trp mutant block to generate W(3Cyto) that has all eight transmembrane Trps replaced. Using W(3Cyto) as the template, the same approach was used to replace the cytoplasmic block of three Trps, W158, W799 and W1104, with degenerate codons and generate a fully Trp-less (WL)-Pgp.

The challenge of the site-saturation mutagenesis approach is the generation of very diverse libraries of mutant DNAs and the requirement for an efficient selection scheme. Thus, to maximize DNA yields we made several procedural improvements. Phusion DNA polymerase (ThermoFisher) was used for optimum PCR fidelity, specificity, and yield. Correct size PCR fragments and linearized vector were gel-purified and the DNA electroeluted into dialysis bags to maximize recovery, and generate sufficient numbers of mutant variants for the second "anneal and fill" PCR reaction. The full-length PCR fragments were ethanol precipitated, and then co-transformed into yeast using the high-efficiency yeast transformation lithium acetate method [4]. To increase transformation efficiency, the transformants were incubated at 30°C in liquid medium for 4-6 hours, allowing homologous recombination [1] and protein expression to occur, before they were plated on uracil-deficient medium to estimate the total number of transformants in each sample.

```
        XhoI                                         NarI
      ~~~~~~~~~                                    ~~~~~~~~~
       L   E   E   N   L   Y   F   Q   G   G   G   A   G   G   S   M   V   S   K   G   E   E   L   F   T
   1  CTC GAG GAA AAT CTT TAC TTT CAA GGA GGT GGC GCC GGT GGA TCT ATG GTT TCT AAA GGT GAG GAA CTG TTT ACT

       G   V   V   P   I   L   V   E   L   D   G   D   V   R   G   H   K   F   S   V   S   G   E   G   E
  76  GGT GTT GTT CCT ATT CTT GTC GAG CTG GAC GGT GAT GTC AGA GGA CAC AAG TTT TCT GTT TCT GGT GAG GGT GAG

       G   D   A   T  N39  G   K   L   T   L   K   F   I   C   T   T   G   K   L   P   V   P   W   P   T
 151  GGT GAC GCC ACC AAC GGA AAA CTG ACC TTG AAG TTC ATT TGT ACT ACT GGT AAG TTG CCT GTT CCT TGG CCT ACT

       L   V   T   T  L64 T65  Y   G   V   Q   C   F   S   R   Y   P   D   H   M   K   R   H   D   F   F
 226  CTT GTC ACT ACC CTG ACT TAC GGT GTT CAG TGT TTT TCC AGA TAC CCT GAT CAC ATG AAA AGA CAT GAC TTC TTC

       K   S   A   M   P   E   G   Y   V   Q   E   R   T   I  S99  F   K   D   D   G  T106 Y   K   T   R
 301  AAG TCT GCC ATG CCA GAG GGT TAC GTC CAG GAG AGA ACT ATC TCT TTC AAG GAC GAT GGT ACT TAT AAA ACT CGT

       A   E   V   K   F   E   G   D   T   L   V   N   R   I   E   L   K   G   I   D   F   K   E   D   G
 376  GCT GAA GTT AAG TTT GAA GGT GAT ACC CTG GTC AAT AGA ATC GAA CTG AAG GGA ATT GAT TTT AAA GAG GAT GGA

       N   I   L   G   H   K   L   E   Y   N  F145 N   S   H   N   V   Y   I  T153 A   D   K   Q   K   N
 451  AAC ATT CTT GGA CAC AAG TTG GAG TAT AAC TTC AAC TCC CAC AAT GTC TAT ATC ACT GCT GAC AAG CAG AAA AAT

       G   I   K  A163 N   F   K   I   R   H   N  V171 E   D   G   S   V   Q   L   A   D   H   Y   Q   Q
 526  GGT ATT AAG GCC AAT TTT AAA ATC CGT CAT AAT GTT GAG GAC GGA TCT GTC CAG CTT GCC GAC CAC TAC CAA CAG

       N   T   P   I   G   D   G   P   V   L   L   P   D   N   H   Y   L   S   T   Q   S  K206 L   S   K
 601  AAT ACT CCA ATC GGA GAT GGT CCA GTC TTG CTG CCT GAC AAT CAT TAT CTG TCT ACC CAG TCT AAG TTG TCC AAG

       D   P   N   E   K   R   D   H   M   V   L   L   E   F   V   T   A   A   G   I   T   L   G   M   D
 676  GAC CCA AAT GAA AAG CGT GAT CAC ATG GTC TTG TTG GAA TTT GTC ACC GCT GCT GGT ATC ACT CTG GGA ATG GAT
                      NarI                                                  NotI
                    ~~~~~~~~~                                            ~~~~~~~~~~~
       E   L   Y   K   G   A   S   G   G   S   W   S   H   P   Q   F   E   K   A   A   A   G   G   G   S
 751  GAG CTT TAC AAG GGC GCC TCT GGA GGT TCC TGG TCC CAC CCA CAG TTT GAG AAA GCG GCC GCC GGC GGT GGT TCT
                                                                                                AgeI
                                                                                              ~~~~~~~
       G   G   G   S   W   S   H   P   Q   F   E   K   G   S   G   H   H   H   H   H   H   *   T   G
 826  GGC GGT GGT TCT TGG TCC CAC CCA CAG TTT GAG AAG GGT TCT GGT CAC CAC CAT CAC CAC CAT TGA ACC GGT
```

Supplemental Figure S2: TEV cleavable, codon-optimized superfolder-GFP sequence. The superfolder-GFP sequence (46 – 762 bp) [5,6] contains the fluorescence enhancing mutations V64L/S65T, and the A206K mutation that minimizes GFP dimerization [7] bolded in red ink, as well as mutations that promote in-vivo protein folding Y39N, F99S, N106T, Y145F, M153T, V163A, and I171V marked in blue ink. Codon usage of the entire gene was optimized following the codon usage table for highly expressed proteins in *S. cerevisiae* and *P. pastoris* yeast as described [8], using the methods of Hunt and Aalberts [9]. An upstream TEV protease site (ENLYFQ) [10], and downstream dual StrepII (WSHPQFEK) and **His$_6$** tags are all spaced out from the GFP by flexible glycine-serine-alanine hinge peptides. Sequences are flanked by *XhoI* and *AgeI* restriction enzyme sites to facilitate cloning, internal site are indicated above the sequence.

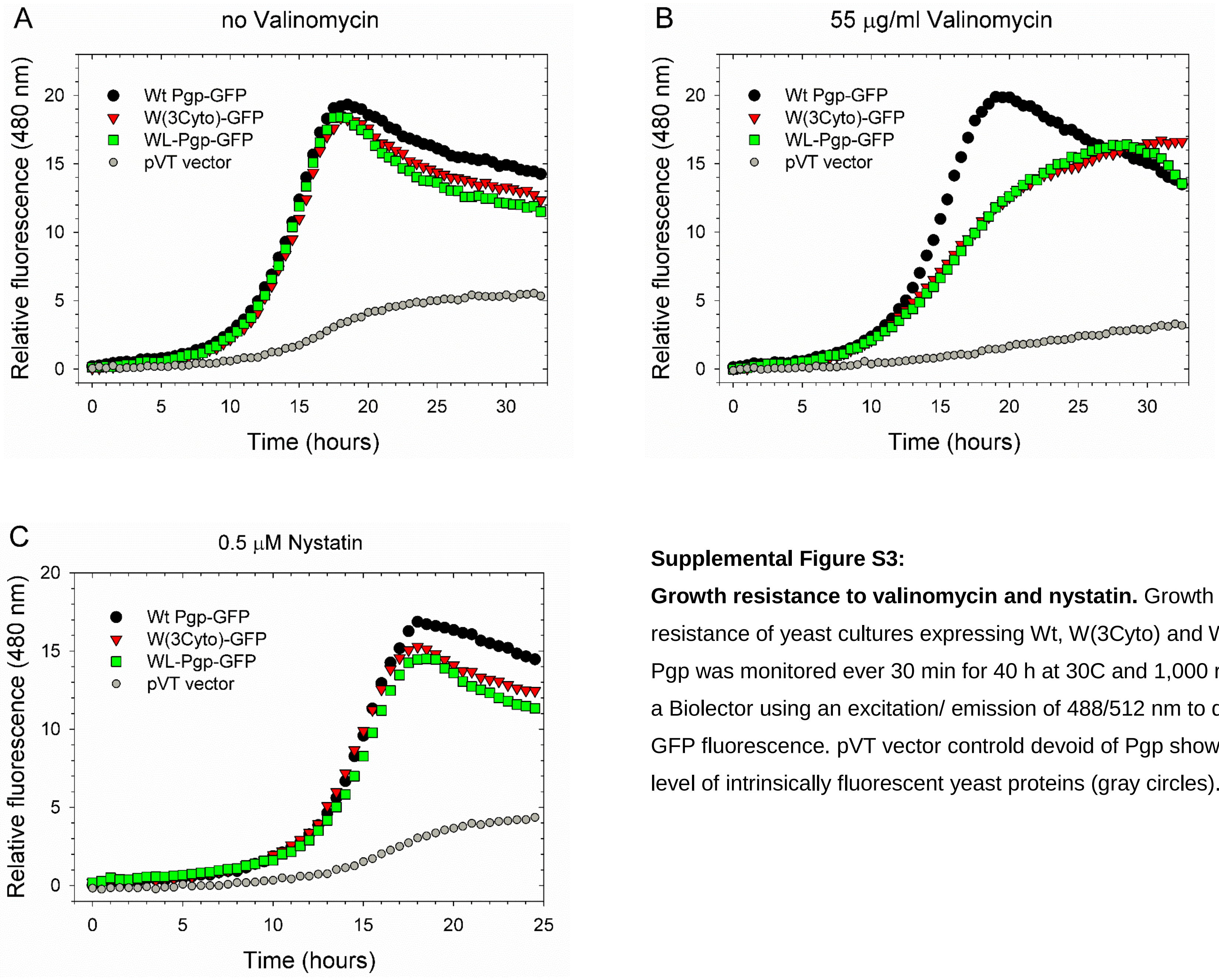

Supplemental Figure S3:

Growth resistance to valinomycin and nystatin. Growth resistance of yeast cultures expressing Wt, W(3Cyto) and WL-Pgp was monitored ever 30 min for 40 h at 30C and 1,000 rpm in a Biolector using an excitation/ emission of 488/512 nm to detect GFP fluorescence. pVT vector controld devoid of Pgp show low level of intrinsically fluorescent yeast proteins (gray circles).

Supplemental Table S1:

Amino Acid Distribution (in %; BLAST 250)

	44	132	158	208	228	311	694	704	799	851	1104
W	53.5	74.9	98.6	100	39.8	83.4	25.4	91.7	92.8	99.7	67.0
G	6.2	0	0	0	0	0	2.6	0.4	0	0	0
A	0.4	3.2	0	0	0	0	3.0	0.4	0	0	0
V	0.4	0	0	0	0.7	0	0.4	1.5	0	0	0
L	0.4	0.7	0	0	1.7	0	26.5	1.1	0	0	0
I	0.4	0	0	0	10.7	0	0.4	2.5	0	0	0
M	0.4	1.1	0	0	9.0	0	1.9	0	0	0	0
P	1.2	0	0	0	0	0	0.4	0	0	0	0
F	0.4	22.0	0.7	0	6.9	0	15.3	0	3.6	0	19.2
Y	0	0.7	0.7	0	9.3	1.0	0	0	3.6	0.3	1.0
S	18.7	0	0	0	0.3	0.3	4.9	0	0	0	0.9
T	3.7	1.1	0	0	0.7	0	10.4	0	0	0	0
C	7.5	0	0	0	3.5	0	0	1.8	0	0	0.9
N	0.4	0	0	0	0	0	0.4	0	0	0	0.3
Q	0	1.8	0	0	6.9	0.3	0	0	0	0	0
H	0	0	0	0	0	0	0	0	0	0	3.1
K	3.3	0	0	0	0	0	0	0	0	0	0.6
R	2.9	0	0	0	1.7	14.8	8.6	0.7	0	0	1.3
D	0	0	0	0	0	0	0	0	0	0	0
E	0	0	0	0	8.7	0	0	0	0	0	0

Amino Acid Distribution (in %; BLAST 1000)

	158	208	851
W	45.7	97.5	98.7
G	0	0	0
A	0	1.1	0.2
V	0	0	0.1
L	0	0	0
I	0	0.2	0
M	0	0	0
P	0	0.9	0.6
F	40.3	0	0
Y	14.0	0.1	0.4
S	0	0	0
T	0	0	0
C	0	0	0
N	0	0	0
Q	0	0	0
H	0	0	0
K	0	0	0
R	0	0	0.2
D	0	0	0
E	0	0	0

In a BLAST [11] search, the 250 (first part) or 1000 (second part) closest relatives to mouse Pgp were analyzed regarding the amino acids found in the position equivalent to the position of the Trps in Pgp.

Supplemental References

1 Kwan, T. & Gros, P. Mutational analysis of the P-glycoprotein first intracellular loop and flanking transmembrane domains. *Biochemistry* **37**, 3337-3350 (1998).

2 Beaudet, L. & Gros, P. Functional dissection of P-glycoprotein nucleotide-binding domains in chimeric and mutant proteins. Modulation of drug resistance profiles. *J Biol Chem* **270**, 17159-17170 (1995).

3 Raymond, M., Gros, P., Whiteway, M. & Thomas, D. Y. Functional complementation of yeast ste6 by a mammalian multidrug resistance mdr gene. *Science* **256**, 232-234 (1992).

4 Gietz, R. D. & Schiestl, R. H. High-efficiency yeast transformation using the LiAc/SS carrier DNA/PEG method. *Nature protocols* **2**, 31-34 (2007).

5 Pedelacq, J. D., Cabantous, S., Tran, T., Terwilliger, T. C. & Waldo, G. S. Engineering and characterization of a superfolder green fluorescent protein. *Nature biotechnology* **24**, 79-88 (2006).

6 Dean, K. M. & Grayhack, E. J. RNA-ID, a highly sensitive and robust method to identify cis-regulatory sequences using superfolder GFP and a fluorescence-based assay. *RNA* **18**, 2335-2344 (2012).

7 Zacharias, D. A., Violin, J. D., Newton, A. C. & Tsien, R. Y. Partitioning of lipid-modified monomeric GFPs into membrane microdomains of live cells. *Science* **296**, 913-916 (2002).

8 Bai, J. *et al.* A gene optimization strategy that enhances production of fully functional P-glycoprotein in Pichia pastoris. *PloS one* **6**, e22577 (2011).

9 Boel, G. *et al.* Codon influence on protein expression in E. coli correlates with mRNA levels. *Nature* **529**, 358-363 (2016).

10 Mohanty, A. K., Simmons, C. R. & Wiener, M. C. Inhibition of tobacco etch virus protease activity by detergents. *Protein Expr Purif* **27**, 109-114 (2003).

11 Altschul, S. F. *et al.* Gapped BLAST and PSI-BLAST: a new generation of protein database search programs. *Nucleic Acids Res* **25**, 3389-3402 (1997).
